# Supplementary figures and images for: Identification and validation of ATP6V1G1-regulated phosphorylated proteins in hepatocellular carcinoma
Source: PLoS One. 2024 Dec 2;19(12):e0310037. doi: 10.1371/journal.pone.0310037 (PMC11611105; doi:10.1371/journal.pone.0310037)

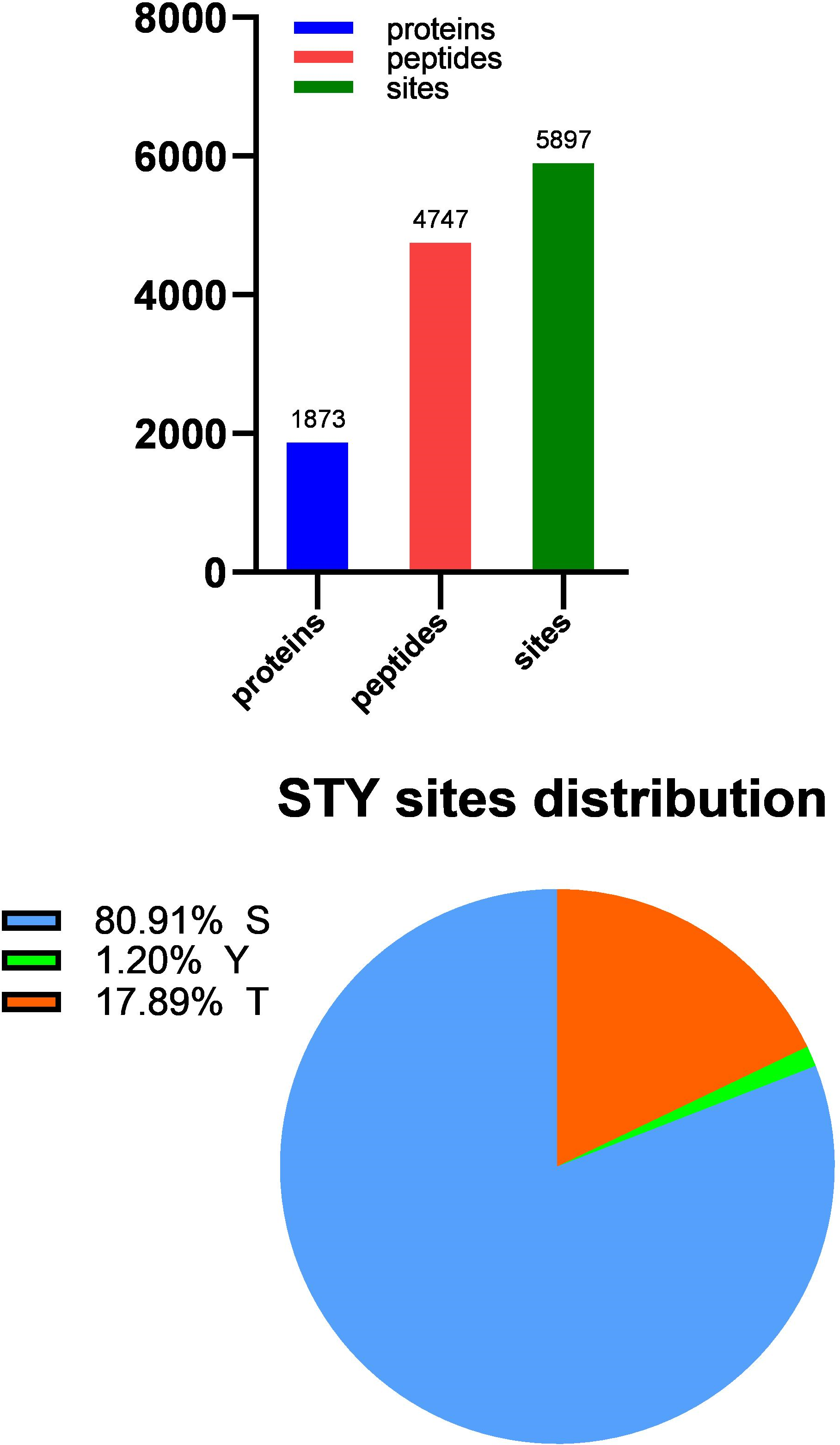

Supplement: S1 Fig — A. Number of proteins, peptides and sites with altered phosphorylation. B. The proportion of threonine, tyrosine and serine amino acid sites changed by phosphorylation. (TIF) [file pone.0310037.s001.tif]

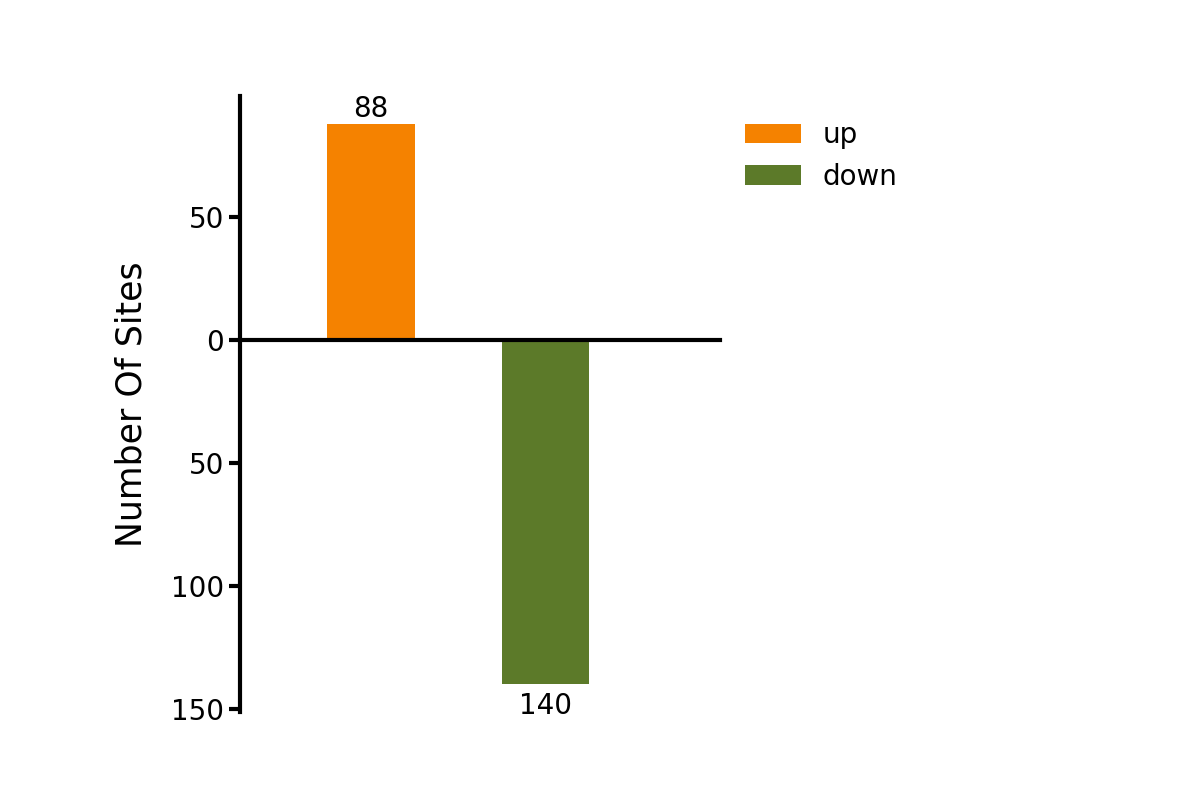

Supplement: S2 Fig — (TIF) [file pone.0310037.s002.tif]

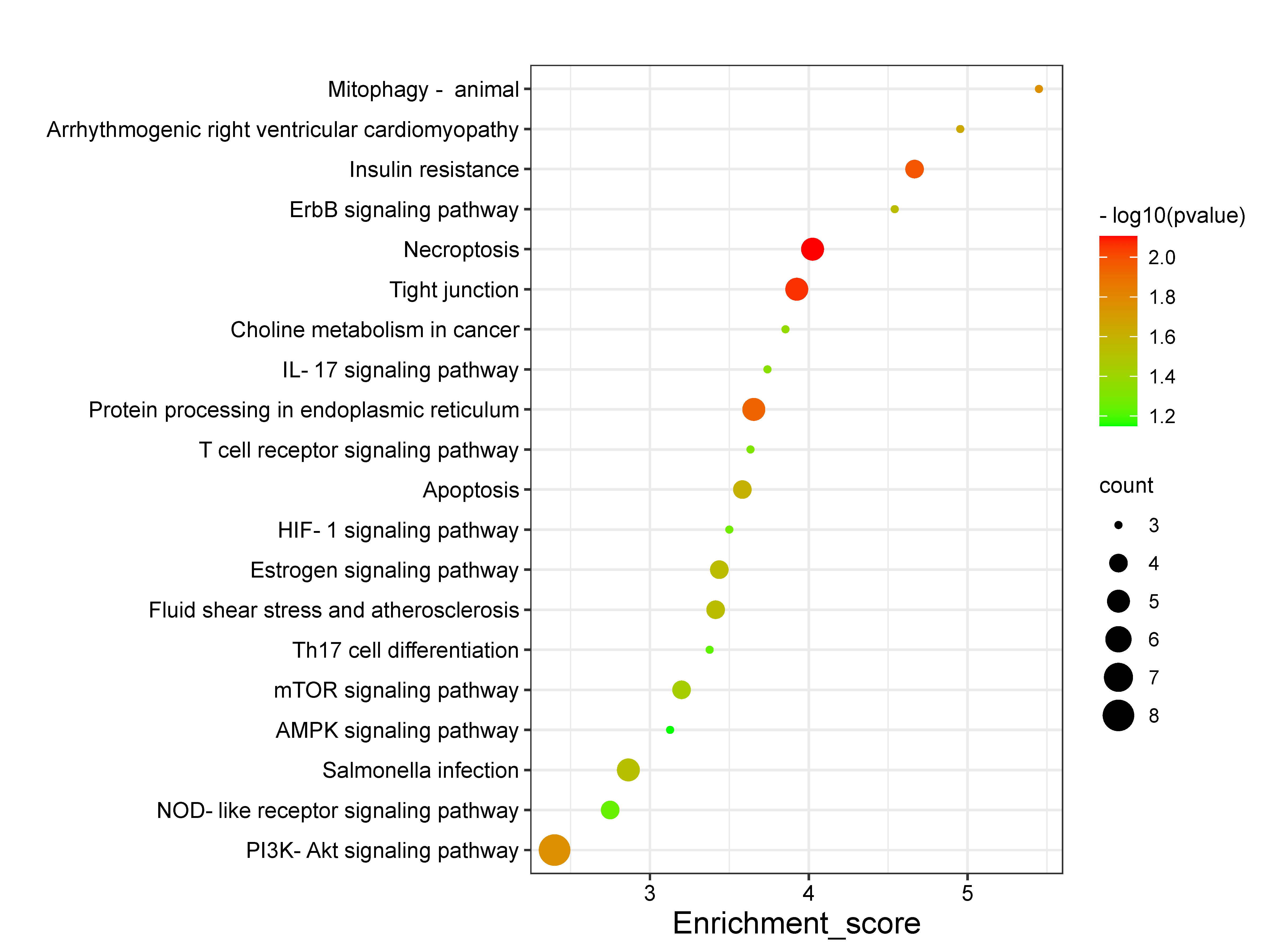

Supplement: S3 Fig — (TIF) [file pone.0310037.s003.tif]

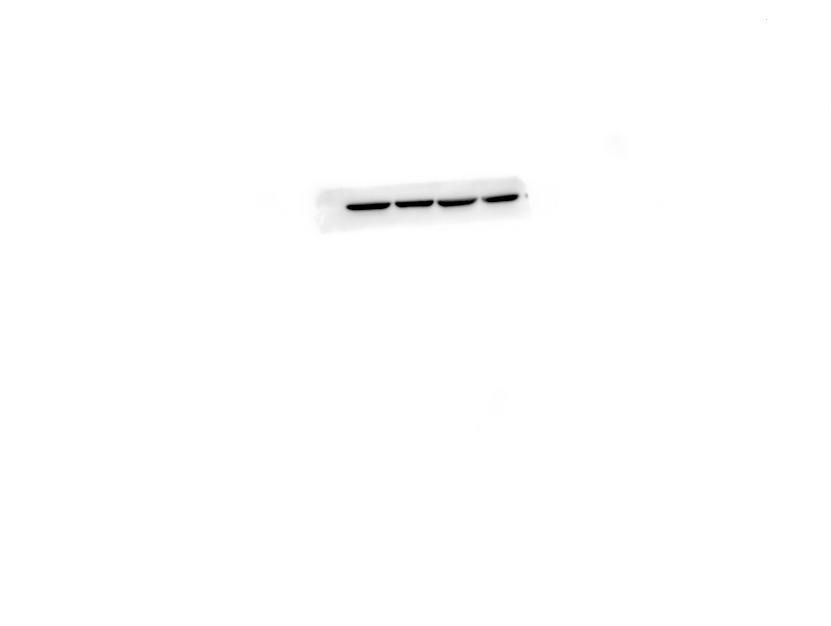

Supplement: S1 File — (ZIP) [file pone.0310037.s004.zip › S1 File/Uncropped western blots-Figure2/WB-actin.tif]

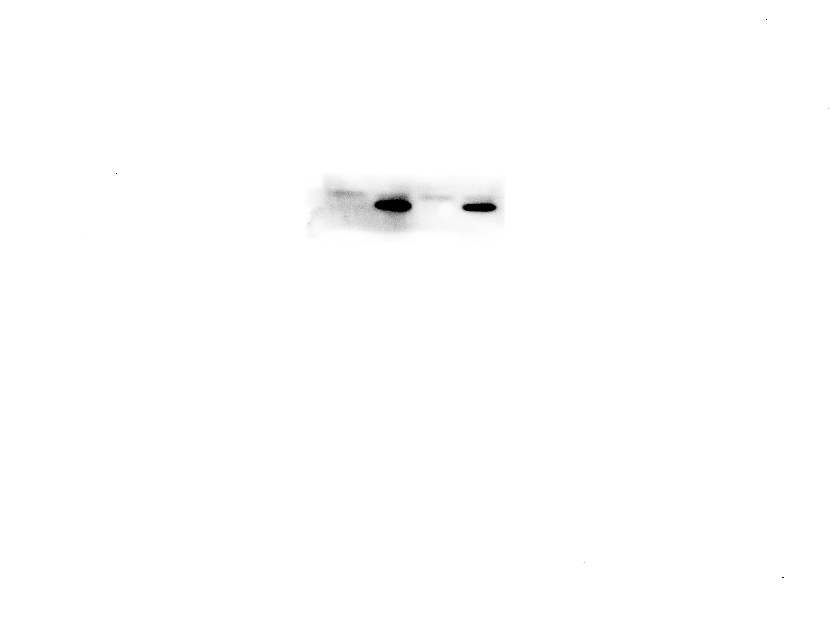

Supplement: S1 File — (ZIP) [file pone.0310037.s004.zip › S1 File/Uncropped western blots-Figure2/WB-flag.tif]

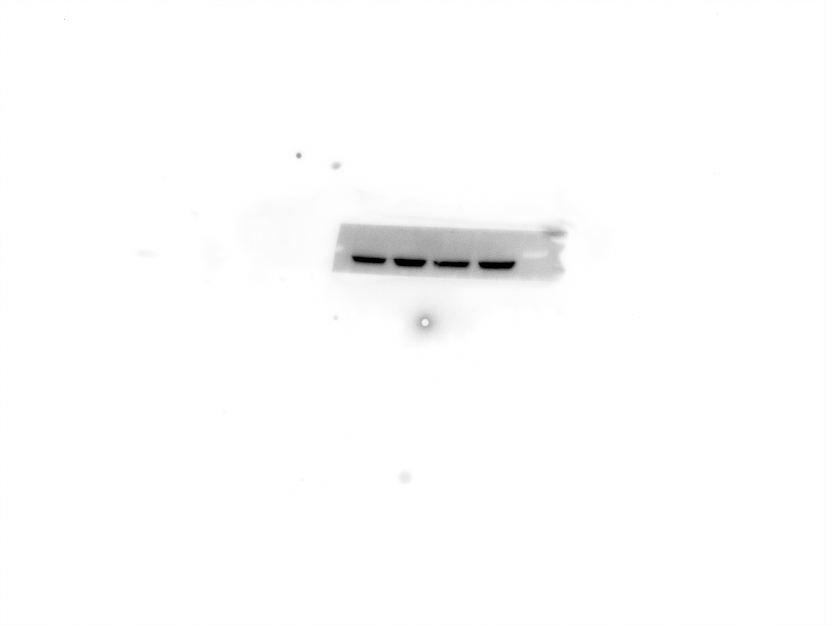

Supplement: S1 File — (ZIP) [file pone.0310037.s004.zip › S1 File/Uncropped western blots-Figure2/WB-V1G1.tif]

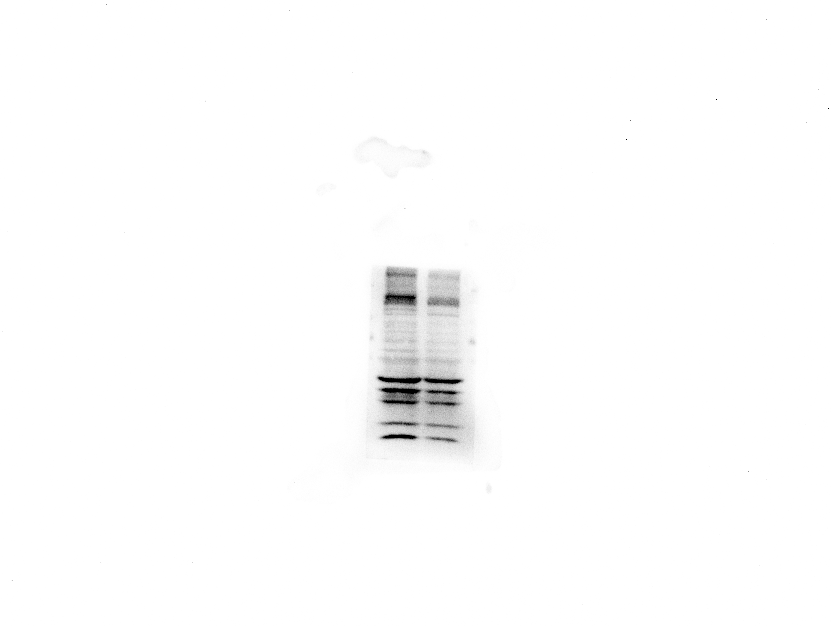

Supplement: S1 File — (ZIP) [file pone.0310037.s004.zip › S1 File/Uncropped western blots-Figure3/WB-p pdk1.tif]

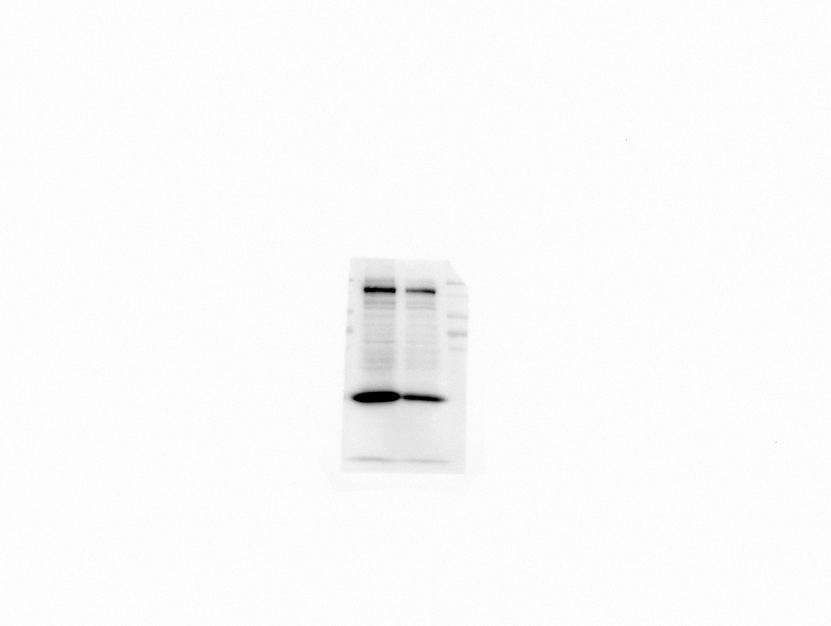

Supplement: S1 File — (ZIP) [file pone.0310037.s004.zip › S1 File/Uncropped western blots-Figure3/WB-p-EEF2.tif]

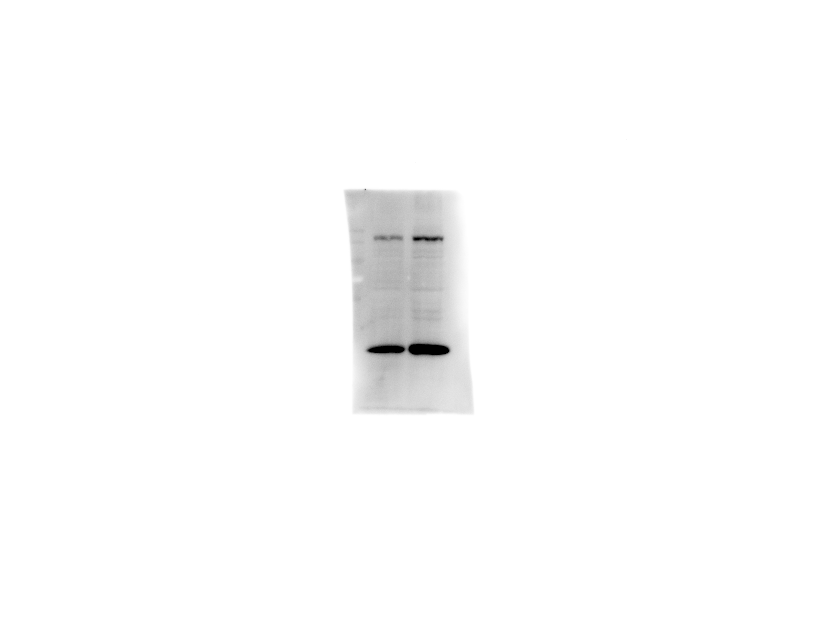

Supplement: S1 File — (ZIP) [file pone.0310037.s004.zip › S1 File/Uncropped western blots-Figure3/WB-P-RPS6.tif]

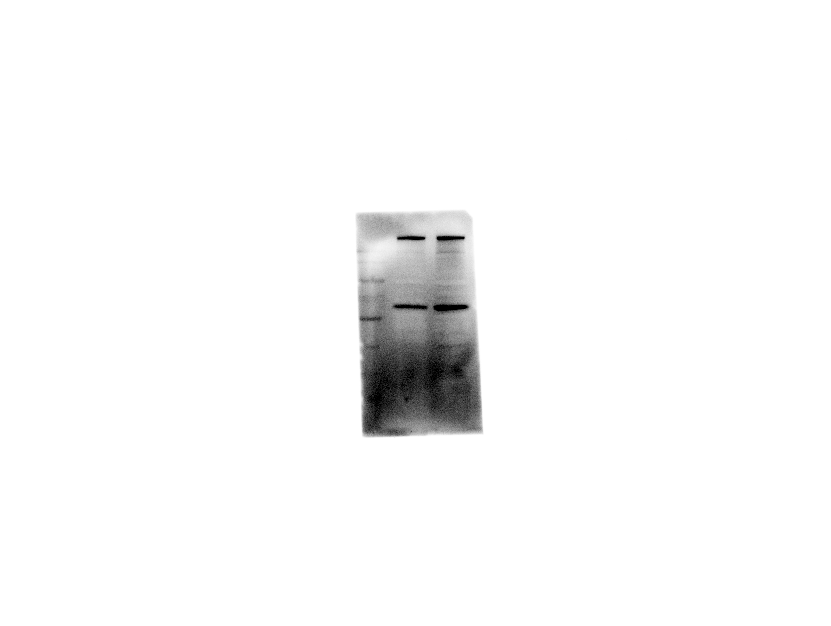

Supplement: S1 File — (ZIP) [file pone.0310037.s004.zip › S1 File/Uncropped western blots-Figure3/WB-p-SQSTM1.tif]

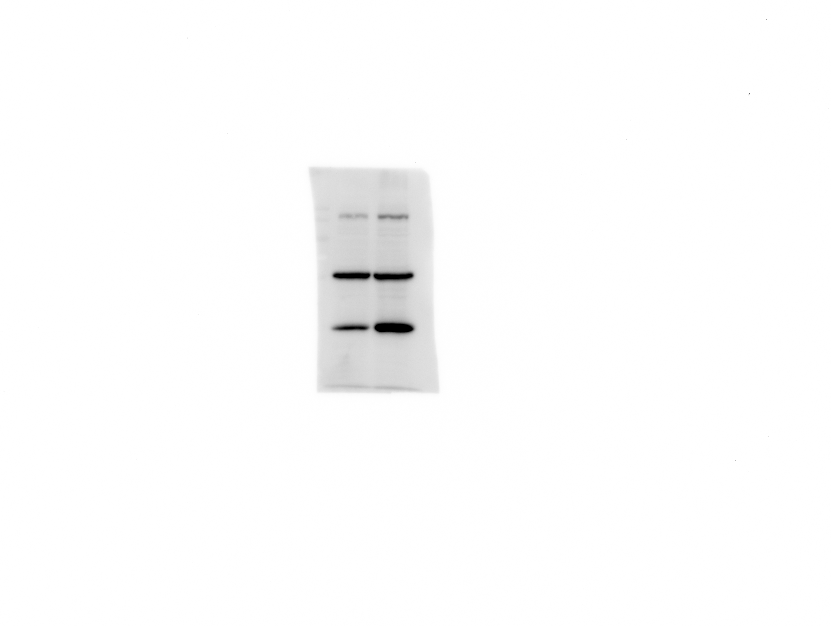

Supplement: S1 File — (ZIP) [file pone.0310037.s004.zip › S1 File/Uncropped western blots-Figure3/WB-a┬-actin.tif]
