# Supplementary material for: Identification and validation of ATP6V1G1-regulated phosphorylated proteins in hepatocellular carcinoma
Source: PLoS One. 2024 Dec 2;19(12):e0310037. doi: 10.1371/journal.pone.0310037 (PMC11611105; doi:10.1371/journal.pone.0310037)
Supplement: Attachment — Submitted filename: Comments.pdf [file pone.0310037.s005.pdf]

The manuscript reported the effect of ATP6V1G1 in HCC and protein expression associated with of ATP6V1G1 activity. The phosphorylated proteins regulated by ATP6V1G1 in HCC were identified by phosphoproteomics and LC-MS/MS analysis. How did the phosphorylated proteins (DEPPs) contribute to the protein expression? How was the impact of ATP6V1G1 on tumor progression deduced based on the present studies? More alternate studies are needed to provide supportive data prior to drawing a convincing conclusion. How do the present results contribute to the mechanistic action of cancer development?
